# Ventricular Fibrillation: Potential Deadly Consequence of Discontinuation of Methimazole Prior to Radioiodine Ablation

**DOI:** 10.7759/cureus.13167

**Published:** 2021-02-06

**Authors:** Billal H Sikandar, Obinna Mmagu

**Affiliations:** 1 Internal Medicine/Cardiology, University of Maryland Prince George's Hospital Center, Cheverly, USA; 2 Cardiology, University of Maryland Prince George's Hospital Center, Cheverly, USA

**Keywords:** methimazole, treatment of hyperthyroidism, qtc prolongation, hyperthyroidism, thyrotoxcosis, anti-thyroid drugs, ventricular fibrillation (vf) storm, heart failure with reduced ejection fraction, r-on-t phenomenon, radioiodine ablation

## Abstract

The following case involves a 62-year-old female patient suffering from heart failure with reduced ejection fraction (HFrEF) secondary to non-ischemic cardiomyopathy and Graves disease, who developed ventricular fibrillation (VF) after discontinuation of methimazole in preparation for radioiodine ablation. Electrocardiogram (ECG) showed a severely prolonged QTc in the setting of thyrotoxicosis, which significantly improved with high dose methimazole. VF secondary to thyrotoxicosis has rarely been reported and the literature review shows scarce data on its mechanism. Our case demonstrates not only a possible mechanism for the arrhythmia, but also highlights a potential risk factor for it. The report details how discontinuing antithyroid medication leads to VF in our patient and reviews the current literature on antithyroid withdrawal prior to radioiodine ablation therapy. Caution should be taken when discontinuing antithyroid medications in patients with advanced heart failure as potentially lethal ventricular arrhythmias can ensue.

## Introduction

Hyperthyroidism imposes various cardiovascular complications and hemodynamic changes like increased contractility, increased preload, and decreased systemic vascular resistance, which all result in increased cardiac output [1,2]. The most common cardiac complication is atrial fibrillation which occurs in approximately 15% of overtly hyperthyroid patients [3]. The occurrence of dangerous arrhythmias such as ventricular fibrillation (VF), although rare, can cause sudden cardiac death [4]. Malignant ventricular arrhythmias secondary to QT prolongation from thyrotoxicosis are seldom encountered in the inpatient setting. Factors that contribute to a fatal arrhythmia are thyrotoxicosis in the setting of an already weakened heart, seen in our patient. Literature review shows that ventricle repolarization is greatly affected in hyperthyroidism [3-5]. Ventricular repolarization is assessed using the QT interval corrected for the heart rate (QTc) on an electrocardiogram (ECG). Research shows a significant positive correlation between QTc and thyroid hormones, especially free T4 levels. However, the mechanism and pathophysiology are not well understood [4-8]. The correlation has even been reported in patients with subclinical hyperthyroidism [7]. Prolonged QTc is associated with an increased risk for lethal arrhythmias such as torsade de Pointes (TdP) or VF. Thyrotoxicosis from antithyroid withdrawal for radioiodine ablation has been reported, however, serious adverse events such as VF have rarely been reported. This case shows after five days of discontinuing methimazole, the QTc increased to dangerous levels, triggering VF in our patient. Various withdrawal periods have been reported: current guidelines dictate that antithyroid medications should be held for three to seven days prior to radioiodine ablation in order to maximize radioiodine uptake [9-13]. However, the literature does not specify the duration of treatment suspension when it relates to individuals with cardiac comorbidities such as advanced heart failure. Five days without methimazole lead to a QTc of 607 ms, which resulted in VF in our patient. Thus, in patients with preexisting heart failure, discontinuation of antithyroid medication should be followed up with careful QTc monitoring prior to radioiodine ablation therapy.

## Case presentation

The patient is a 62-year-old female with heart failure with reduced ejection fraction (HFrEF) from nonischemic cardiomyopathy (ejection fraction of 25%), automatic implantable cardioverter-defibrillator (AICD), hypertension, and Graves’ disease who presented after an episode of syncope at home. The patient stated that she was standing by her dresser when she suddenly started having palpitations, diaphoresis, and nausea. After that, she lost consciousness for a few seconds and fell to the floor (no head trauma). She denied any prodromal dizziness, light-headedness, or vertigo. The patient was diagnosed with Graves’ disease nine months prior to presentation. The patient stated that she was instructed by her endocrinologist to discontinue her methimazole for one week as she was scheduled to undergo elective radioiodine ablation of the thyroid. A recent thyroid uptake study showed 83% homogeneous uptake in the thyroid, consistent with graves thyrotoxicosis. The patient presented to the hospital five days after discontinuing her methimazole. She also reported palpitations, poor appetite, feeling anxious, and fatigue since stopping her medication. Home medications included aspirin 81 mg daily, metoprolol succinate ER 25 mg daily, sacubitril/valsartan 24/26 mg twice daily, spironolactone 25 mg daily, furosemide 40 mg daily, and methimazole 20 mg daily. The patient denied any past surgical history except for AICD placement. Social and family histories were significant only for Graves' disease in biological mother.

**Figure 1:**
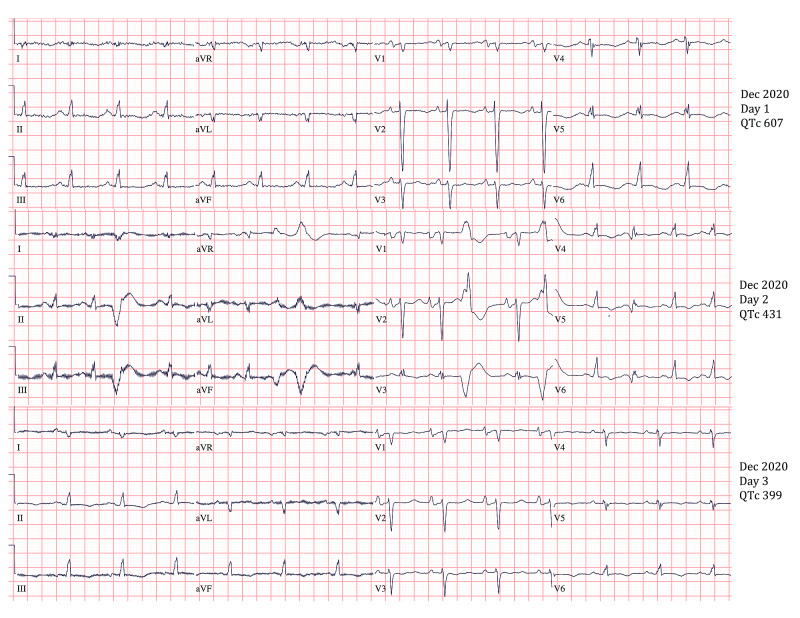
QTc Improvement With Methimazole Treatment Image shows improving QTc interval after treatment with high-dose methimazole (30 mg every 6 hours). After >24 hours of treatment, QTc interval improved from 607 to 431 and after >48 hours of treatment QTc improved to 399. QTc: corrected QT interval for heart rate.

**Figure 2:**
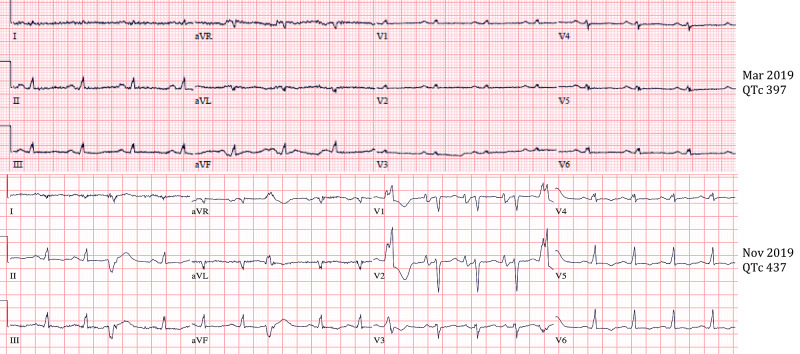
Relationship Between QTc and Free T4 Image shows a positive correlation between QTc interval and the patient’s free thyroxine (T4). In March 2019, free T4 was 2.08 and QTc was 297 ms. In November 2019, free T4 increased to 4.70 and QTc increased to 437 ms.

**Figure 3:**
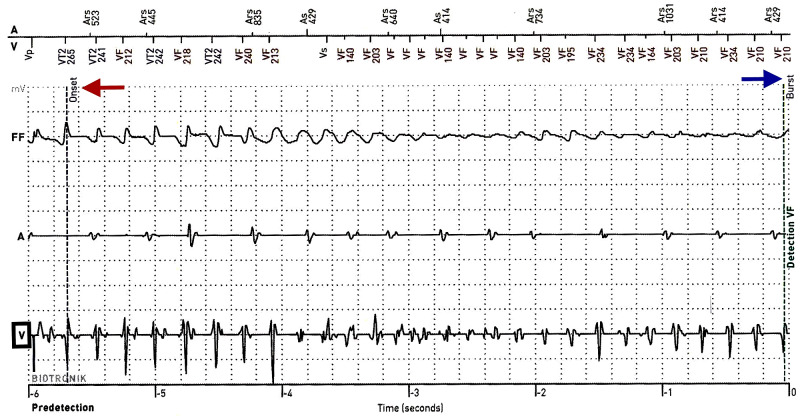
Ventricular Fibrillation on AICD Interrogation AICD interrogation revealed VF, which can be seen on the ventricular electrogram (V) on the AICD (black box). The onset of the VF is labeled with the blue arrow and the red arrow signifies the 40-Joule defibrillation shock, which converted the patient to sinus rhythm. VF: ventricular fibrillation; FF: far-field electrogram; AV: marker channel; V: ventricular electrogram; A: atrial electrogram; AICD: automatic implantable cardioverter-defibrillators.

**Figure 4:**
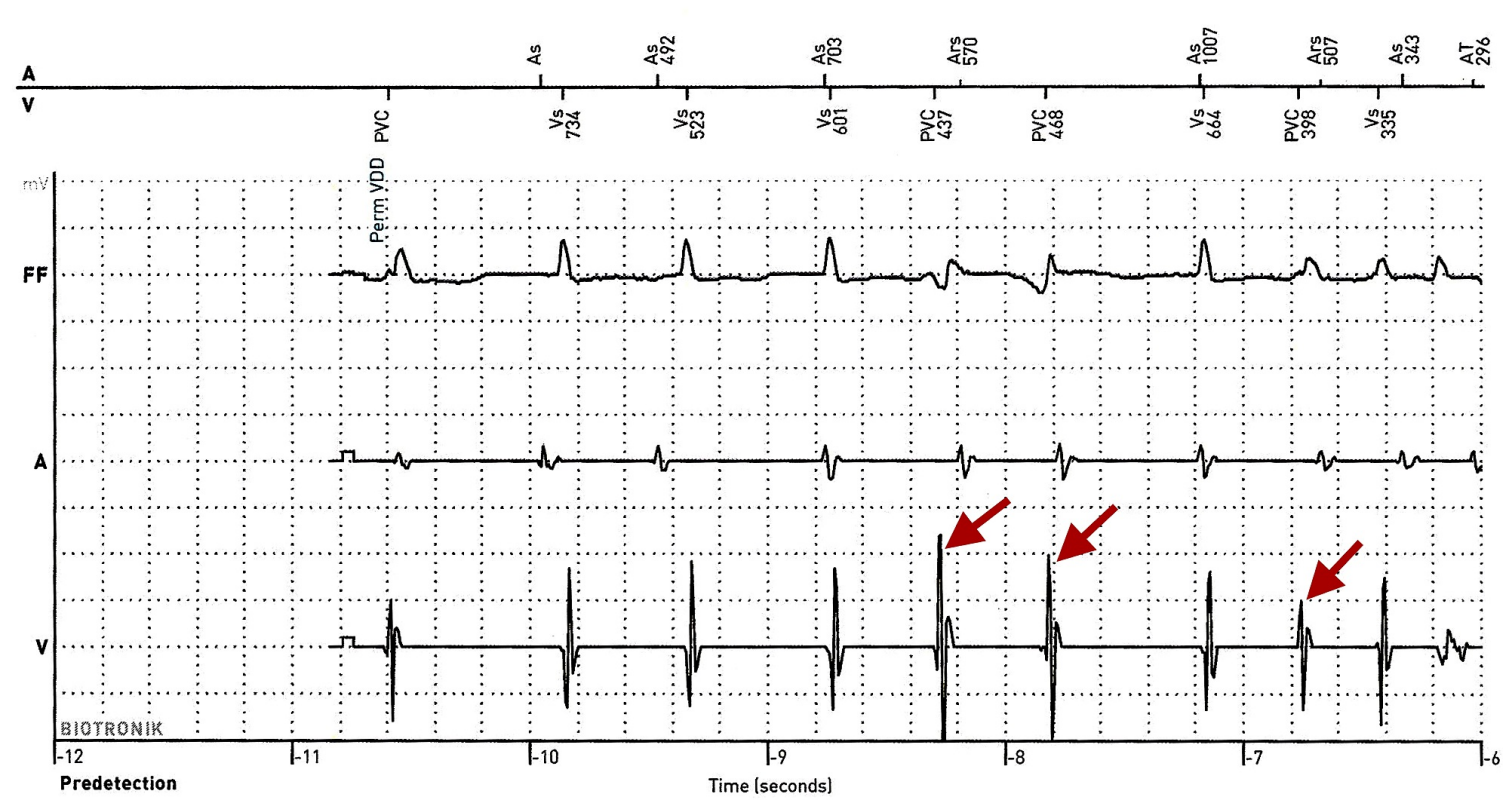
PVCs Prior to Ventricular Fibrillation: R-on-T Phenomena AICD detected PVCs prior to VF. PVCs (red arrows) can be seen on the ventricular electrogram (V). These PVCs supports the assumption that along with QTc prolongation, the PVCs could have resulted in the “R on T” phenomena, which triggered the VF. FF: far-field electrogram; AV: marker channel; V: ventricular electrogram; A: atrial electrogram; PVC: premature ventricular contraction.

On admission, patient vitals were a pulse of 90 (regular), blood pressure of 104/58, respiratory rate of 18, and oxygen saturation of 100% on room air. Physical examination was unremarkable. Patients labs were significant for TSH of less than 0.005 (reference range 0.270-4.200 mcIU/mL), free T3 of 21.3 (reference range 0.20-4.40 pg/mL), and free T4 of greater than 7.77 (reference range 0.90-1.71 ng/dL). chest X-ray was negative for acute pathology. Initial ECG showed nonspecific intraventricular conduction delay and severe QTc prolongation of 607 ms (Figure 1). Medical record review showed patient recently followed up with a primary care physician who obtained thyroid studies and ECG. The patient’s QTc interval three weeks prior to admission was 437 (Figure 2). Thyroid function testing showed TSH of less than 0.005, free T4 of 4.7, and free T3 of 8.80. During her visit, methimazole was increased from 10 mg daily to 20 mg daily. Thus, the patient’s QTc increased from 437 ms to 607 ms (Figures 1 and 2) after discontinuing methimazole for five days. AICD interrogation revealed the patient had an episode of VF (same time as syncope occurred) and received an appropriate shock (Figure 3). AICD also showed frequent premature ventricular contractions (PVCs) prior to VF (Figure 4). Although not apparent on our patient’s AICD interrogation, the PVCs could have fallen on a T-wave from a previous contraction enabling the “R-on-T” phenomenon, which possibly triggered her VF [14]. The patient was found to have frequent PVCs on both AICD and in-hospital ECGs (Figures 1 and 4). Patient’s magnesium on admission was 2.1 (reference range 1.7-2.6 mg/dL) and potassium was 3.9 (reference range 3.5-5.1 mmol/L). Troponin and Pro-BNP were within normal limits. Transthoracic echocardiogram (TTE) showed a stable ejection fraction of 25%, no acute wall motion abnormalities, and no new structural pathologies when compared to the previous TTE done six months ago. Therefore, the likelihood of an electrolyte abnormality, myocardial ischemia or infarct, or new structural abnormality as the cause of VF was less likely. Further record review traced the patient’s diagnosis of hyperthyroidism to nine months ago, where she had a QTc of 397 (Figure 2). Thyroid studies showed TSH <0.005, free T4 of 2.08, and normal free T3 at 4.05.

Endocrinology was consulted and recommended the initiation of methimazole at a dose of 30 mg every six hours. EP Cardiology also recommended the initiation of antithyroid medication, avoidance of QT-prolonging drugs, maintenance of potassium above 4 and magnesium above 2, and serial ECG for QTc monitor. On day 2 of admission, and after receiving a high dose of methimazole for 24 hours (onset of action 12-16 hours), repeat ECG showed QTc of 431 (Figure 1). On day 3 of admission, QTc was 399 (Figure 1). The patient was discharged home on 30 mg methimazole twice daily with instructions to follow up with her endocrinologist and cardiologist within one week. A follow-up with the patient revealed that radioiodine ablation was postponed until the patient became euthyroid; discussion regarding potential lifelong antithyroid medication instead of ablation was being considered. Post-discharge AICD interrogation did not reveal any further ventricular arrhythmias.

## Discussion

Thyroid hormone whether in its absence or excess seems to be able to cause QTc prolongation. The mechanism by which both hypothyroidism and hyperthyroidism can cause ventricular repolarization delay/OTc prolongation is not well understood. Literature review shows that in the case of hypothyroidism, there could be a combination of disturbances leading to QTc prolongation. Studies show that in hypothyroidism, there is an alteration of autonomic modulation of the heart leading to sympathovagal imbalance and an increased inhomogeneity of ventricular recovery times [8,15,16]. Furthermore, in a study conducted on guinea pigs, there was a decrease in the slow component of delayed rectifier K current (IKs), believed to be a major factor in disrupting ventricular repolarization [16,17].

Numerous studies have shown a strong association between hyperthyroidism and QTc prolongation. A positive correlation between free T4 and the degree of QT prolongation was seen; patients with higher levels of free T4 had longer QTc duration [1,3-8]. This positive correlation was present with our patient: as free T4 increased, QTc also increased (Table 1). Normalization of free T4 leads and achievement of the euthyroid state has been shown to normalize QTc duration in hyperthyroid patients [4]. Although repeat-free T4 was not done in our patient after high dose methimazole treatment, serial QTc did show significant and continued improvement (Figure 1 and Table 1). The pathophysiology of QTc prolongation due to a hyperthyroid state is not well understood. A few studies and proposed theories have explained the possible mechanism by which thyroid hormone increases ventricle repolarization and thus QTc. One theory focuses on the effect of thyroid hormone on the cardiac myocyte Na/K1 ATPase receptor. It is proposed that increased activity in this receptor secondary to the action of T4 leads to increased intracellular potassium, resulting in membrane hyperpolarization and higher QTc interval [4,5,8]. A separate study done on rats showed increased gene expression of the Na/K1 ATPase receptor after transplanted hearts were treated with T4 [4]. These findings suggest that there is a possible dose-dependent effect of T4 on the intracellular potassium of cardiac myocytes and, in turn, ventricle repolarization manifests as prolonged QTc [4]. Although very rare, new-onset thyrotoxicosis can precipitate VF theoretically by inducing coronary vasospasm leading to myocardial ischemia [1]. However, no evidence of ischemia was noted in our patient as she did not complain of angina; cardiac enzymes were within normal limits throughout admission; ECG did not show evidence of ischemia; TTE was negative for acute wall motion abnormality.

Patients with HFrEF and Graves' disease are at higher risk for cardiac arrhythmias than patients without heart failure. Maladaptive hypertrophic and fibrotic myocardial remodeling in advanced heart failure can predispose a patient to a spectrum of ventricular arrhythmias [1]. Discontinuation of medications such as methimazole in a patient with advanced heart failure can further increase the risk for ventricular arrhythmias. Our patient was instructed to discontinue medications in order to decrease the risk of radioiodine ablation failure. A meta-analysis of 14 randomized controlled trials with a total of 1306 participants showed that the continuation of antithyroid drugs in the week prior to radioiodine ablation increased the rate of ablation failure [13]. The efficacy of radioiodine therapy was reduced when the antithyroid medication was continued during ablation. Research shows that not only did radioiodine have a lower uptake and shorter half-life, but also there was a varied distribution of the radioiodine throughout the thyroid [12].

The minimum duration for withdrawal of treatment, prior to radioiodine ablation, has not been clearly established. The World Journal of Nuclear Medicine and the Society of Nuclear Medicine recommend three-day withdrawal as it has been shown to be effective in treating Graves’ disease without exacerbating hyperthyroidism. Yet, controlled clinical trials and comparative studies show that a two-day withdrawal is sufficient to restore the efficacy of radioiodine for thyroid ablation [9-12]. In the comparative study, patients in a two-day antithyroid withdrawal regimen were examined prospectively using radioiodine uptake, serum-free T4, and an outcome of therapy. These parameters were compared to those in a seven-day withdrawal regimen retrospectively. The results showed no statistically significant difference in radioiodine uptake in the two-day withdrawal group compared to the seven-day withdrawal group. The mean serum-free T4 measured 24 hours after radioiodine therapy revealed that the seven-day group had significantly higher levels than the two-day group. Thus, this study shows that the two-day withdrawal period had similar results for uptake and did not exacerbate hyperthyroidism when compared to the seven-day withdrawal period [10]. In the controlled clinical trial, radioiodine kinetics was studied under continued thiamazole medication and after discontinuation for one to two days in 316 patients. The results showed that when the antithyroid medication was discontinued for at least two days, radioiodine uptake was near normal; however, uptake within one day of discontinuation was reduced. Patients in the continued thiamazole medication group showed a decreased uptake by a factor of 2.5 [12].

A two-day withdrawal period may be useful for high-risk patients with cardiac comorbidities such as advanced heart failure. In our patient, discontinuation of methimazole for two days instead of seven days could have prevented thyrotoxicosis and in turn VF from QTc prolongation. It is evident from our patient's ECGs that serum T4 levels had a positive correlation with QTc interval (Table 1). Severely elevated T4 can lead to dangerously prolonged QTc resulting in ventricular arrhythmias such as VF or TdP. The risk of prolongation of QTc is likely further increased in our patient given her advanced heart failure. Withdrawal of treatment should be minimized and if indicated to the least number of days. QTc should be monitored frequently when the withdrawal is indicated, especially regarding patients with cardiac comorbidities. Further research and concrete guidelines for the duration of withdrawal of antithyroid medication prior to radioiodine ablation in high-risk patients is needed.

**Table 1:** Positive Correlation Between Correct QT Interval and Free Thyroxine The table shows a positive correlation between QTc and free T4 levels in our patient. As free T4 levels increased the QTc of our patient also increased. The QTc interval dramatically decreased from 607 ms to 431 ms after receiving >24 hours of high dose methimazole (30 mg every six hours). Free T4 was not checked after initial testing during the December 2020 admission. Corrected QT interval (QTc) is in milliseconds; free thyroxine level (T4) is in nanograms per deciliter.

	March 2019	November 2019	December 2020: Day 1	December 2020: Day 2	December 2020: Day 3
Corrected QT interval	397	437	607	431	399
Free thyroxine level	2.08	4.7	>7.77	Not checked	Not checked

## Conclusions

In this case, the patient’s syncopal episode due to VF may have been due to severe QT prolongation in the setting of thyrotoxicosis from medication withdrawal. Given her history of nonischemic cardiomyopathy with an ejection fraction of 25%, the risk for ventricular arrhythmias is already elevated. Caution should be taken when withdrawing antithyroid medication in patients with advanced heart failure as the risks for severe QTc prolongation and subsequent malignant ventricular arrhythmias may be increased. If a withdrawal is indicated, the least amount of duration should be chosen and frequent monitoring of QTc should be performed.
